# Meeting the challenge of genomic analysis: a collaboratively developed workshop for pangenomics and topological data analysis

**DOI:** 10.1093/bioadv/vbae139

**Published:** 2024-09-27

**Authors:** Haydeé Contreras-Peruyero, Shaday Guerrero-Flores, Claudia Zirión-Martínez, Paulina M Mejía-Ponce, Marisol Navarro-Miranda, J Abel Lovaco-Flores, José M Ibarra-Rodríguez, Anton Pashkov, Cuauhtémoc Licona-Cassani, Nelly Sélem-Mojica

**Affiliations:** Centro de Ciencias Matemáticas, UNAM, Antigua Carretera a Pátzcuaro # 8701, Residencial San José de la Huerta, Morelia, Michoacán 58089, Mexico; Centro de Ciencias Matemáticas, UNAM, Antigua Carretera a Pátzcuaro # 8701, Residencial San José de la Huerta, Morelia, Michoacán 58089, Mexico; School of Engineering and Sciences, Tecnológico de Monterrey, Monterrey 64849, Mexico; Biology Department, Duke University, Durham, NC 27708, United States; School of Engineering and Sciences, Tecnológico de Monterrey, Monterrey 64849, Mexico; Genetic Engineering, Research and Advanced Studies Center (CINVESTAV-Irapuato), Irapuato 36824, Mexico; Genetic Engineering, Research and Advanced Studies Center (CINVESTAV-Irapuato), Irapuato 36824, Mexico; C3 Idea, León, Guanajuato 37500, Mexico; National School of Higher Studies Morelia UNAM, Morelia 58089, Mexico; School of Engineering and Sciences, Tecnológico de Monterrey, Monterrey 64849, Mexico; Centro de Ciencias Matemáticas, UNAM, Antigua Carretera a Pátzcuaro # 8701, Residencial San José de la Huerta, Morelia, Michoacán 58089, Mexico

## Abstract

**Motivation:**

As genomics data analysis becomes increasingly intricate, researchers face the challenge of mastering various software tools. The rise of Pangenomics analysis, which examines the complete set of genes in a group of genomes, is particularly transformative in understanding genetic diversity. Our interdisciplinary team of biologists and mathematicians developed a short Pangenomics Workshop covering Bash, Python scripting, Pangenome, and Topological Data Analysis. These skills provide deeper insights into genetic variations and their implications in Evolutionary Biology. The workshop uses a Conda environment for reproducibility and accessibility. Developed in The Carpentries Incubator infrastructure, the workshop aims to equip researchers with essential skills for Pangenomics research. By emphasizing the role of a community of practice, this work underscores its significance in empowering multidisciplinary professionals to collaboratively develop training that adheres to best practices.

**Results:**

Our workshop delivers tangible outcomes by enhancing the skill sets of Computational Biology professionals. Participants gain hands-on experience using real data from the first described pangenome. We share our paths toward creating an open-source, multidisciplinary, and public resource where learners can develop expertise in Pangenomic Analysis. This initiative goes beyond advancing individual capabilities, aligning with the broader mission of addressing educational needs in Computational Biology.

**Availability and implementation:**

https://carpentries-incubator.github.io/pangenomics-workshop/

## 1 Introduction

The rapid evolution of technology demands continuous development of new skills for effective data analysis in Biology. This is especially important for multidisciplinary professionals in Computational Biology who must keep up with the latest advancements. In the landscape of Genomics data, researchers often need to be proficient in multiple programming languages and various domain-specific software tools. Recognizing the urgency of adapting to this dynamic field, developing educational materials in Computational Biology is crucial. These materials are pivotal in ensuring accessibility to complex topics, fostering interdisciplinary education, and preparing students to tackle current and future challenges. Multidisciplinary didactic material provides individuals with the tools to grasp the nuances of Genomics data analysis, encouraging the integration of diverse skills and knowledge.

Communities of Practice and Short-Format Training (SFT) offer opportunities to acquire foundational skills in multidisciplinary areas (Teal *et al.* 2015, Magnano *et al.* 2022). Various studies outline guidelines for designing (Devenyi *et al.* 2018, Williams *et al.* 2023) and implementing (Ponsero *et al.* 2020) effective SFT workshops to optimize their impact on trainees. One of these communities is The Carpentries, which aims to introduce scientists to good coding practices (Wilson *et al.* 2014), while providing materials for applying the learned code and acquiring new tools in specific domains. The Carpentries Incubator platform provides communities with resources for creating didactic content. Essential steps include establishing a community around the lesson, promoting modularity, and educating contributors on its development. We named our community of practice “The Collaborative Community for Genomic Bacterial Analysis and Practice.”

Pangenomics, a multidisciplinary field relevant to both biologists and mathematicians, explores gene family content variation within closely related organisms (Tettelin *et al.* 2005). Mathematics has played a pivotal role in developing algorithms to define gene families and analyze their growth tendencies. Topological Data Analysis (TDA), a new branch of Mathematics, offers insights into genomic patterns and deviations from hierarchical evolutionary processes (Emmett and Rabadan 2014; Prantzalos *et al.* 2024), such as recombination or Horizontal Gene Transfer (HGT). Efforts are underway to develop tools that apply TDA to Pangenomics, benefiting mathematicians, biologists, and data scientists by enhancing their research pipelines.

We developed a comprehensive four-lesson SFT workshop adhering to the curriculum design guidelines offered by The Carpentries (Becker and Michonneau 2020). The workshop covers introductory programming in two coding languages, Bash and Python, and software-based pangenome construction and visualization. Finally, it explores TDA in understanding gene family evolution. Our goal is to foster catalytic learning, inclusivity, and community building, thereby facilitating lesson maintenance, workshop scaling, and diversification of our audience. We anticipate continuous refinement of this workshop, envisioning it as a valuable resource for new instructors and a comprehensive guide for those initiating Pangenomics analyses from scratch.

## 2 Story of the workshop

**Figure 1.**
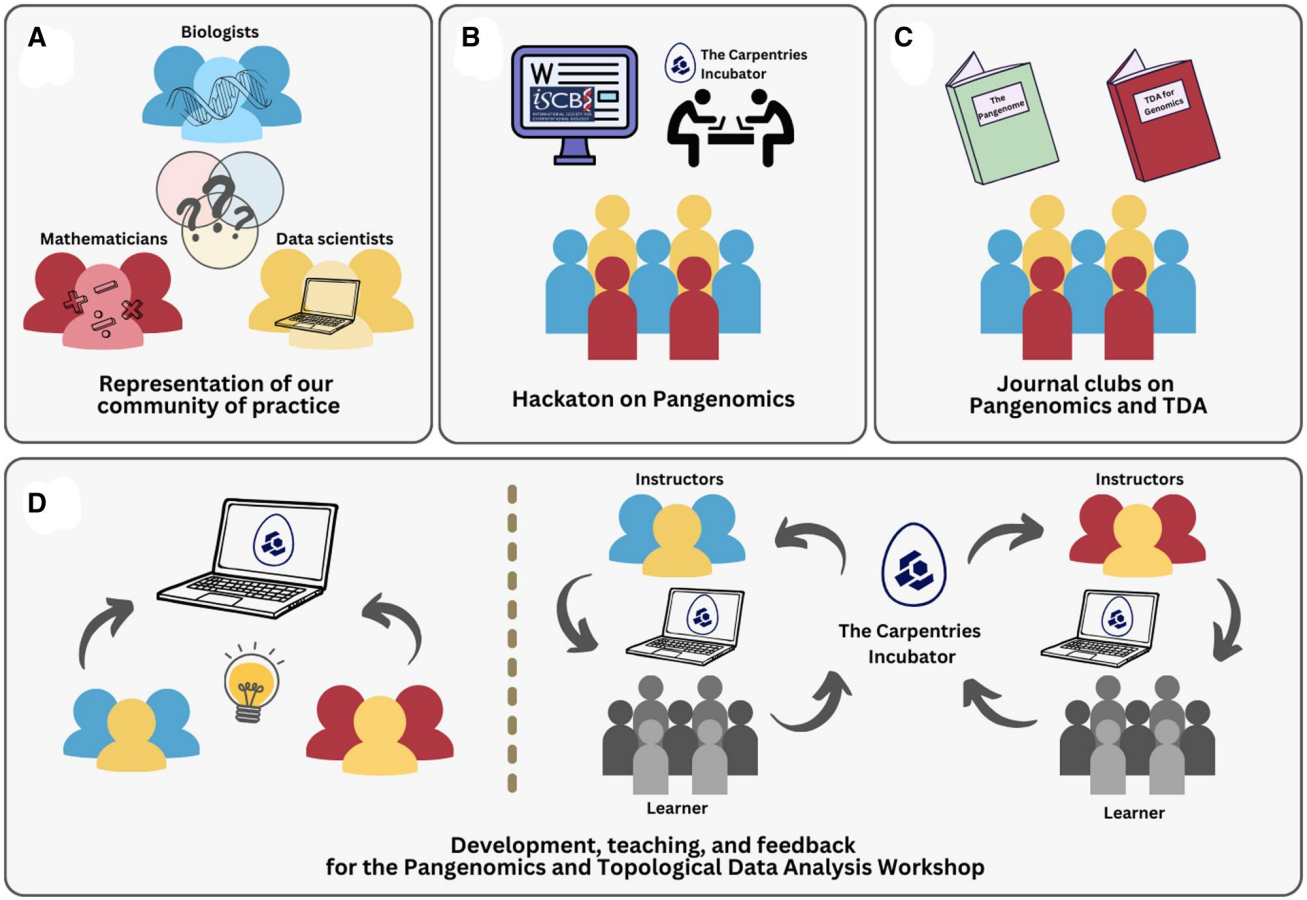
Collaborative efforts within the Pangenomics Workshop development process. (A) Community of practice from diverse disciplines engaging in learning Pangenomics. (B) Active participation in the ISCB-Wikipedia contest and internal hackathons to create the Pangenomics Workshop. (C) Engagement in journal club sessions to promote multidisciplinary learning. (D) The workshop was polished based on community feedback and will continue to improve within the Pangenomics and Topological Data Analysis Workshop teaching process. The Carpentries Incubator logo was included in this figure.

Our developer’s team of the Pangenomics Workshop initiated their story together in 2020 with the development of the Metagenomics Workshop (Zirión-Martínez *et al.* 2024), which is the first community-developed workshop in The Carpentries Incubator accepted in The Carpentries Lab. After this experience, our research topics shifted toward Pangenomics, an area in which we previously lacked expertise. This resulted in creating a bigger community of practice comprised of researchers, postdoctoral fellows, undergraduate and graduate students, and principal researchers from various institutions (UNAM, Tecnológico de Monterrey, CINVESTAV, and Duke University) and backgrounds (Mathematics, Data Science, and Biology) (Fig. 1A). The community became interested in researching and facilitating the conceptual and practical understanding of Pangenomics.

First, we participated in the 10th International Society of Computational Biology (ISCB) Wikipedia Contest. Since 2007, the ISCB organized this annual contest to improve the quality of Wikipedia articles on Computational Biology (O’Neill *et al.* 2017). The pangenome article won the 10th ISCB Wikipedia contest in 2021, increasing in size from 21 616 bytes to 47 244 bytes after the competition. Our community also won the first Latin America Wikipedia ISCB Contest with the Spanish version of the article. We noticed a significant gap during our review of bioinformatic tools for Pangenomics analyses, which we intended to use in our research and for the Wikipedia article. Although there are excellent user manuals (Eren *et al.* 2015; Gautreau *et al.* 2020; Bruno and Pablo 2019) and tutorials for specific tools (Murat Eren 2021; Murat Eren *et al.* 2016), as well as comprehensive literature (Tettelin and Medini 2020), these resources primarily focus on how to run the software or the theory. However, they do not introduce basic concepts or provide short lessons to quickly bring students with no prior programming knowledge up to speed for Pangenomics analysis. There were no public SFT materials that covered both theory and practice for beginners.

This, and our previous successful experience with The Carpentries Incubator (Zirión-Martínez *et al.* 2024), motivated us to create a Pangenomics Workshop. We started the development with a week-long hackathon where we brainstormed and started deciding and sketching what we wanted to include (Fig. 1B). The mathematicians in our group saw an opportunity to analyze pangenomes using resources from their area of expertise by applying TDA to study HGT, which is involved in the evolution of pangenomes. As a result, we broadened our scope and created a multidisciplinary workshop.

To homogenize and deepen our understanding of the topics we wanted to include in the lessons, we organized two journal clubs (Fig. 1C). To read and comment, the first one, on the Tettelin and Medini’s book on the pangenome (Tettelin and Medini 2020), and a second one on Rabadán and Blumberg’s (2020) text on TDA and Genomics ().

An important part that motivated the creation of this workshop was the difficulty we experienced when starting research in Pangenomics. We struggled to achieve a comprehensive overview of a pangenome pipeline, encompassing the identification of input data, file formats, preprocessing requirements, and output files. Additionally, we encountered challenges in selecting appropriate software for various purposes, whether for generating interactive outputs or handling hundreds of genomes. The mathematicians faced particular difficulties with terminology, as certain terms, such as “homology,” have different meanings in Mathematics and Biology. For this reason, we began by developing the materials that included the theoretical background and the tools we needed in our research and that we were already learning. From there, we expanded the contents to include the necessary episodes to create a coherent and complete flow of knowledge. We focused on including all the steps for a complete pipeline by thinking about how our research would have benefited from having those materials to start learning from.

During the episode’s development, our community formed small teams to study each tool we wanted to teach. We then made draft versions of the episodes and had the rest of the community study the draft to test whether the knowledge provided was enough and whether the code was working and is easy to follow (Fig. 1D). When improving the episodes, we focused on making the materials appealing and understandable to the group’s biologists and mathematicians. The tools we encountered while learning how to perform Pangenomics analyses that were unnecessary or redundant for our minimal pipeline, were included as Other Resources to give the learners more options and a broader view of the current Pangenomics research.

After completing the initial versions of our Pangenomics and TDA lessons, we began teaching them and receiving feedback (Fig. 1D) (see Section 5.2). The final and ongoing phase of the development of SFT online workshops is the continuous maintenance and teaching of the materials. This includes updating the content when the methods become obsolete and receiving user feedback. Comments are primarily collected via GitHub issues from other instructors who use the materials and their learners, as well as the broader community interested in the lessons.

## 3 Workshop description

We developed online materials for a workshop aimed at biologists, mathematicians, and other data scientists. The workshop aims to give them the skills to confidently run a pipeline for annotating genomes, building a pangenome, and analyzing it with TDA. The website is available at: Https://carpentries-incubator.github.io/pangenomics-workshop/.

Our workshop has a modular design that caters to audiences with or without previous coding skills. The online materials include a Learning Guide that assists both independent learners and instructors in selecting appropriate modules and exercises based on the learner’s background. The workshop incorporates modern pedagogical approaches, such as interactive learning, practical examples, and a standardized episode structure to make learning more effective and engaging for individual learners and instructor-led in-person or online workshops.

### 3.1 Lesson 1: introduction to the command line

**Figure 2.**
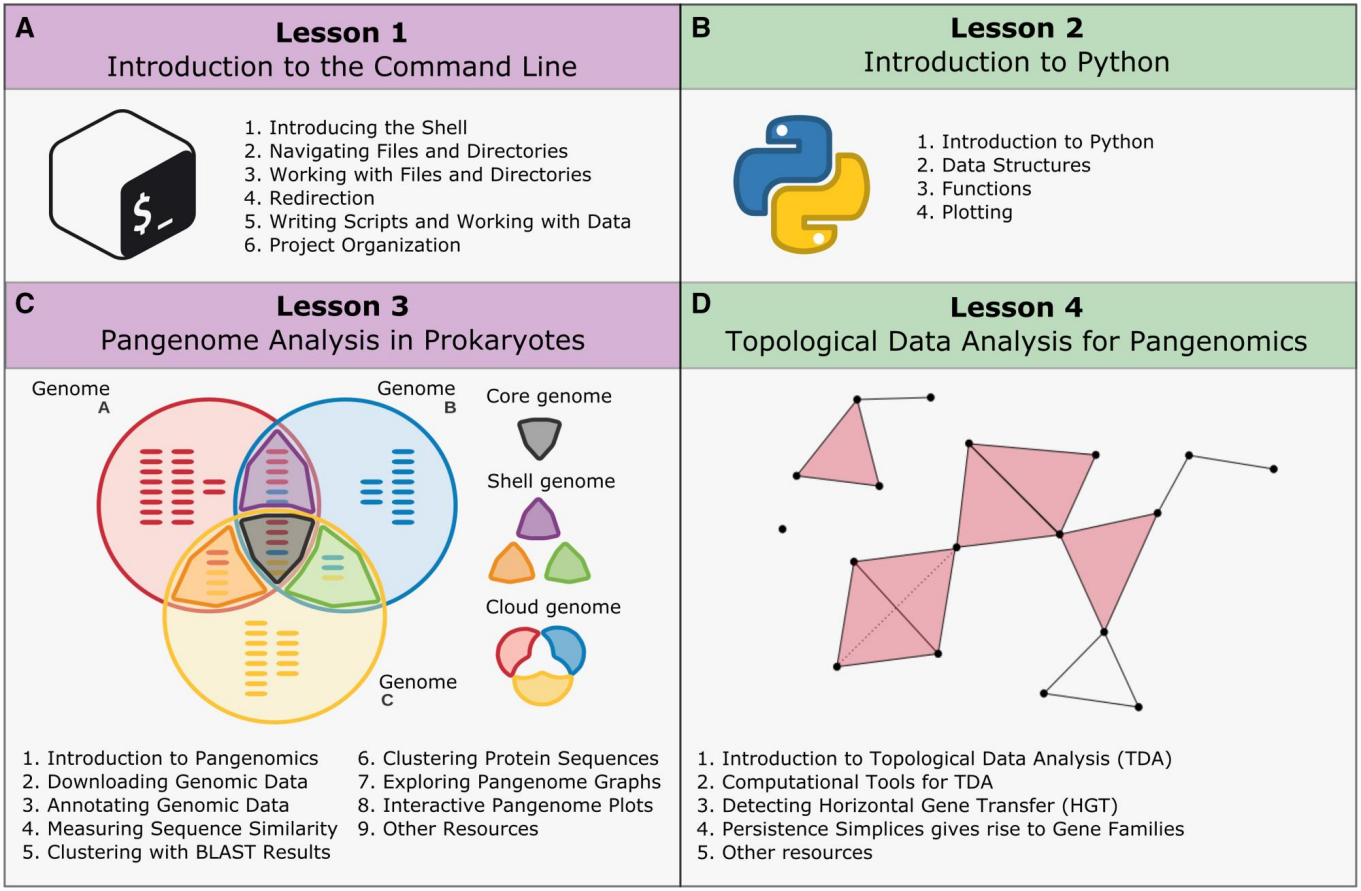
The general structure of the Pangenomics Workshop. The workshop includes four lessons divided into several episodes: (A) Introduction to the Command Line (03:45 h), (B) Introduction to Python (02:45 h), (C) Pangenome Analysis in Prokaryotes (05:40 h), and (D) Topological Data Analysis for Pangenomics (03:44 h). The episodes covered in each lesson are indicated.

This lesson (Fig. 2A) aims to teach coding language Bash to learners without prior programming experience. It is based on the material of the Genomics Workshop of Data Carpentry (Becker *et al.* 2019). It covers an introduction to the shell, connecting to remote machines, navigating the file system, working with files (with examples from the functional annotation files needed for Pangenomics), and making coding more efficient with loops, scripts, and sound project organization practices.

### 3.2 Lesson 2: introduction to Python

Python and R stand out as two primary coding languages for data analysis. Since the episodes of lesson 4 on TDA were developed using the Python library Gudhi, including the essentials of Python programming became necessary. The episodes of this Introduction to Python Lesson (Fig. 2B) encompass manipulating Python’s data types and structures, declaring and utilizing functions, writing conditional statements, optimizing code with loops, and visualizing data. While this Python lesson is concise and focused on facilitating TDA knowledge acquisition, learners seeking a deeper understanding of Python are encouraged to two more comprehensive Python lessons from The Carpentries (Bostroem *et al.* 2016; Lee *et al.* 2018). R programming language also offers packages for TDA, similar to those used in Python. The Other Resources chapter in lesson 4: Https://github.com/tdaverse/tdaverse provides the link to a collection of R lessons.

### 3.3 Lesson 3: pangenome analysis in prokaryotes

Pangenomics aims to understand the diversity of genes in a group of genomes. One objective is to distinguish between gene families shared by the group members and those specific to some of them (Tettelin and Medini 2020). Several programs can accomplish this in distinct ways, answering different biological questions. Although many tools have excellent usage tutorials(Vernikos 2020), we were unaware of a comprehensive educational resource that unifies Pangenomics understanding and discusses the diverse available tools. In this lesson, as shown in Fig. 2C, we benefit from the skills learned in the previous Introduction to the Command Line Lesson to analyze eight *Streptococcus agalactiae* genomes. These data, selected as a case study, revisit the original genomes studied by Tettelin *et al.* (2005), who observed gene families’ intraspecies variation and proposes the pangenome concept (Medini *et al.* 2020).

This Pangenomic lesson is composed of nine episodes. In Episode 1, an introduction to Pangenomics concepts is provided. Episode 2 presents a pipeline that includes downloading public genomes to enable their analysis. Episode 3 includes the functional annotation of the genomes downloaded in Episode 2. Episodes 4 and 5 introduce the basics of gene family clustering. Finally, three open-source programs that allow the analysis of different pangenome properties are presented in Episodes 6–8: GET HOMOLOGUES, PPanGGOLiN, and Anvi’o, respectively. Table 1 describes the complete list of episodes. At the end of this lesson, we suggest exploring other resources and analyses, such as Meta-pangenomics (Ma *et al.* 2020).

**Table 1.** Episodes from the “Pangenome Analysis in Prokaryotes” lesson.

Time	Episodes	Questions
**00:00–00:25**	1. Introduction to Pangenomics	What is a pangenome?
**00:25–01:10**	2. Downloading genomic data	How to download public genomes by using the command line?
**01:10–02:15**	3. Annotating genomic data	How can I identify the genes in a genome?
**02:15–02:55**	4. Measuring sequence similarity	How can we measure differences in gene sequences?
**02:55–03:30**	5. Clustering with BLAST Results	How can we use the blast results to form families?
**03:30–04:10**	6. Clustering protein sequences	Can I cluster my sequences automatically?
**04:10–04:50**	7. Exploring pangenome Graphs	How can I build a pangenome of thousands of genomes?
**04:50–05:20**	8. Interactive pangenome plots	How can I obtain an interactive pangenome plot?
**05:20–05:40**	9. Other Resources	What can I do after I have built a pangenome?

### 3.4 Lesson 4: TDA for pangenomics

TDA is a branch of Mathematics that explores data shapes using tools from algebraic topology, such as persistent homology, Betti numbers, and Euler characteristics. This approach is particularly useful in Pangenomics to understand complex genomic structures and variations. It is well known that genes are usually transmitted from parents to offspring through vertical inheritance. However, in microorganisms, genes can also be transmitted from one individual to another that is not its offspring and might not even be of the same species. This phenomenon, called HGT, is a significant factor that shapes pangenomes by introducing genetic diversity.

TDA has been shown to accurately describe the patterns and dynamics of HGT in microorganisms (Rabadán and Blumberg 2020). In our workshop, the TDA lesson (Fig. 2D) revisits the annotations of antimicrobial resistance genes in *S. agalactiae* pangenome, providing clear evidence of HGT. This example not only illustrates the utility of TDA in identifying and understanding HGT but also naturally intertwines the content covered throughout the workshop, reinforcing the importance of TDA in comprehensive Pangenomics analysis.

In the TDA lesson, the primary objects of analysis are simplicial complexes. These objects generalize graphs, where vertices are called 0-simplices, edges as 1-simplices, triangles as 2-simplices, and so on. A collection of n-simplices adhering to specific rules forms the simplicial complex. For a more detailed explanation, refer to Dey and Wang (2022). These simplices are formed by varying a similarity parameter between data points. Examples of this parameter include the Hamming distance between genomes or sequence identity between genes. As we change this parameter, we obtain a collection of simplices over the range of similarity, creating a filtration associated with the simplicial complex. Persistent homology measures the persistence of these simplices as the distance parameter varies.

Pangenomics seeks to elucidate how gene families are shared among a group of organisms. Persistent homology can detect the presence of 1-holes in the simplicial complexes, which indicate genetic cycles or loops, suggesting the occurrence of HGT (Rabadán and Blumberg 2020) or revealing pangenomes with families that deviate from the typical vertical inheritance pattern. By considering the persistent simplices at a given parameter, we can describe the pangenome associated with that similarity parameter. In other words, we vary the sequence identity to determine if a gene belongs to a certain gene family or another.

In Episode 1, TDA is introduced, and mathematical tools such as simplicial complexes and persistent homology are described. Episode 2 focuses on the computational tools required to compute the persistent homology of a simplicial complex. Three different methods for constructing a simplicial complex are explored, along with the procedure to obtain the associated persistent diagrams and barcodes. Episode 3, explores the use of persistent homology to study crucial aspects of Evolutionary Biology, emphasizing phylogenetic representation and vertical inheritance. This episode underscores the powerful synergy between Biology and Mathematics in uncovering complex patterns in species evolution. Furthermore, it guides the detection of HGT in *S. agalactiae* resistance genes. Episode 4 presents the procedure to compute the simplicial complex associated with a mini-genome file and obtain the persistence families of genes that make up the pangenome at different similarity parameters between genes. Refer to Table 2 for a comprehensive list of episodes and their corresponding questions.

**Table 2.** Episodes from Topological Data Analysis for Pangenomics lesson.

Time	Episode	Questions
**00:00–00:50**	1. Introduction to Topological Data Analysis	What is topological data analysis?
**00:50–01:35**	2. Computational Tools for TDA	How can I computationally manipulate a simplex?
**01:35–02:35**	3. Detecting Horizontal Gene Transfer	How can I detect HGT with TDA?
**02:35–03:25**	4. Persistence Simplices give rise to Gene Families	How can I apply TDA to describe pangenomes?
**03:25–03:45**	5. Other Resources	What other tools are available in TDA?

## 4 Short format training strategies

The workshop comprises four lessons spread over approximately sixteen hours (Tables 1 and 2 and Fig. 2). One-third of the workshop duration (5 h and 45 min) is allocated to exercises graded according to difficulty levels: 26 for beginner, 18 for intermediate, and 5 for advanced; see supplementary Tables S1–S4. This practical part includes live coding, hands-on exercises, and active learning activities. To implement the training, we considered three teaching axes: Before the workshop, we prepared the cloud setup and standardized the episodes; during the workshop, we followed teaching strategies; and after the workshop, we ensured lesson maintenance. The material is readily available on various operating systems and includes introductions to Biology, Computing, and Mathematics, making it accessible to researchers from diverse backgrounds. We have designed a training program that aims to be inclusive of different communities, promotes catalytic learning, and is sustainable in the long term.

### 4.1 Inclusivity is considered before the workshop

**Figure 3.**
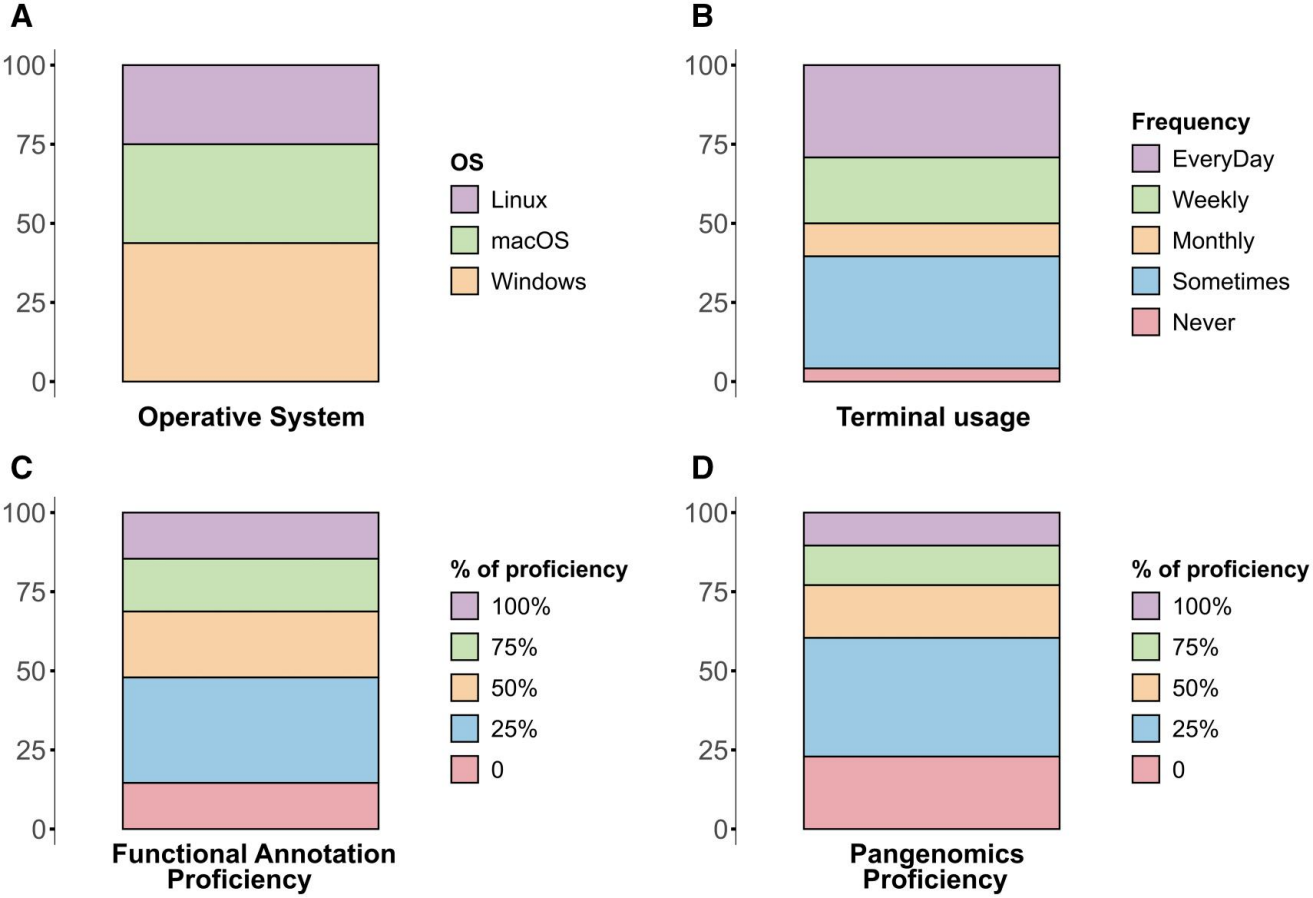
Pre-workshop survey results: Percentage of participants in each response category. (A) Participants are divided almost equally among the three main operating systems: Linux, Windows, and Mac. (B) The frequency of terminal usage is variable, with only a few participants who never use it. (C and D) A larger percentage of participants have higher proficiency in annotation than Pangenomics. The questions asked to the participants were as follows: (A) What operative system do you use on your computer? (B) How frequently do you use the terminal? (C) Rank your functional annotation proficiency before the workshop, and (D) Rank your Pangenomics proficiency before the workshop.

Participants are expected to possess diverse levels of computational, biological, mathematical, and bioinformatic skills. Pre-workshop surveys are conducted to assess these proficiencies before the workshop. This, along with the Learning Guide page in the online materials, enables instructors to determine if certain episodes or lessons should be omitted or taught with additional emphasis. Our investigation encompasses a spectrum of factors, including disparities in operating systems, command line skills, and familiarity with Pangenomics concepts. We interrogated the participants about what operating system they use (Fig. 3A) and the frequency with which they interact with the terminal or command line (Fig. 3B). Lastly, participants are prompted to assess their proficiency in genomic functional annotation and Pangenomics on a scale ranging from 0% to 100%, depicted in Fig. 3C and D.

#### 4.1.1 Cloud setup

To establish a consistent learning environment and teaching experience, we have implemented remote computing infrastructure. This setup minimizes obstacles for students unfamiliar with technical installations, ensuring sufficient computing capability regardless of their configurations. The only requirement for students is a computer with internet access and a terminal installation. Free server access is offered upon request for workshops; self-learners can request temporary accounts. Servers are provided by The Collaborative Community for Genomic Bacterial Analysis and Practice and the sub-community The Carpentries Mexico for individuals choosing to follow the lessons on a different computer, step-by-step instructions for configuring remote machines and an alternative installation with complete Conda environments are provided on the setup page. Additionally, the Docker container aapashkov/panworkshop is available in Dockerhub and has an image of all the workshop dependencies.

#### 4.1.2 Standardized episodes

Following The Carpentries’ instructional design, each episode begins with a set of learning objectives and guiding questions. The content covers explanations of concepts and code, along with formative exercises. Every episode ended with clear summaries of the knowledge gained.

### 4.2 Catalytic learning is promoted during the workshop

The workshop promotes catalytic learning—learning that facilitates self-directed learning after the workshop (Williams *et al.* 2023)—by providing sufficient knowledge to ensure learners feel confident in understanding future content independently. Lessons include supportive tools that encourage self-learning, such as formative exercises, guidance on seeking help through forums or tutorials, and suggestions for additional content for continued learning. Additionally, workshop attendees and self-learners are guaranteed at least 6 months of access to the virtual instance for continued learning, even with their own data, within a storage limit of 25GB.

#### 4.2.1 Live-coding

Programming simultaneously with the students offers a practical coding experience where participants learn the code itself, the coding practices of the instructors, and how to solve errors as they emerge. Exercises and small group discussions enable learners to solve problems with their peers, encourage participation, and reinforce their new knowledge and skills.

#### 4.2.2 Content review

At the end and beginning of each session, learners are asked to review, list, and explain the content covered in previous episodes. A collaborative document is used for simultaneous written and spoken reviews to reaffirm the material and highlight topics that need reinforcement.

#### 4.2.3 Exercises

We offer exercises of varying difficulty levels, including conceptual and practical exercises, to foster self-learning for both beginners and advanced students. Since learners come from different backgrounds, we introduce programming concepts with practical examples, such as for-loops for genome annotation.

### 4.3 Sustainability is promoted after the workshop

#### 4.3.1 Sustainability

One workshop goal is to ensure that learning materials remain available, usable, relevant, and reliable (Williams *et al.* 2023). The creators of this lesson belong to two groups involved with bioinformatics education: A student chapter of the International Society of Computational Biology and the sub-community The Carpentries Mexico. In both groups, we frequently participate in external workshops as participants or trainers. These activities help us to connect with others and promote the use of the workshop’s material. This continuous revision of the lessons promotes its sustainability. Additionally, we can receive public feedback on the Issues GitHub site https://github.com/carpentries-incubator/pangenomics/issues.

## 5 Improving future workshops

### 5.1 Teaching experience

We started teaching the workshop for the Bioinformatics class at the National University Autonomous of México (UNAM) for the students in the Information Technology for Science Major and graduate students in Mathematics interested in Biology. We sought external opportunities after having the first teaching experiences and including their feedback in the episodes. We taught the pangenome lesson four times: In 2022 at the first Latin American Genome Mining Workshop for 20 participants, at the ISCB’s Latam in 2022 for 5 participants, at the 2nd Summer Retreat on Bioinformatics and Complex Networks in 2023 for 20 participants, and through The National Bioinformatics Node of the Center for Genomic Sciences (NNB-CCG) in January 2024, reaching up to 50 participants from Latin America. The pangenome plus the TDA material were taught two times, at the EMALCA in 2023 and in the Annual Biomathematics Autumn School, for 40 and 20 people, respectively. We had two instructors and one assistant per five participants in these workshops. Having taught the workshop externally and incorporating participant input, we are in the alpha stage as defined by The Carpentries (Becker and Michonneau 2020).

### 5.2 Receiving feedback

When we teach the lessons, we conduct post-workshop surveys among the participants and keep the lessons open, allowing us to improve them based on their feedback. When we ran the workshop emphasizing the TDA lessons for 40 undergraduate students from a mathematical background in the EMALCA, the students expressed that they enjoyed the TDA course as it provided a good approach to applying theoretical mathematical concepts in practice. They also mentioned that it was a valuable introduction to Pangenomics and Genomics, leaving them with potential ideas for further exploration. It was also mentioned that the course was easy to follow, educational, and interesting. The majority of the students’ comments were positive, and they appreciated understanding how both disciplines are interconnected. However, some students did not like it as much since they felt it resembled a programming course. This helped us to understand that we needed to work on clarifying the expectations of the course.

We received comments about the workshop at the 2nd Summer Retreat on Bioinformatics and Complex Networks and The National Bioinformatics Node of the Center for Genomic Sciences (NNB-CCG). Students and professors of related genomic areas pointed out a disconnection between the Pangenomics and TDA lessons. To better connect the material we included antimicrobial resistance annotation in the Pangenomics lesson and showed in the TDA lesson how the topological persistence diagram of antimicrobial resistance gene families includes holes.

**Figure 4.**
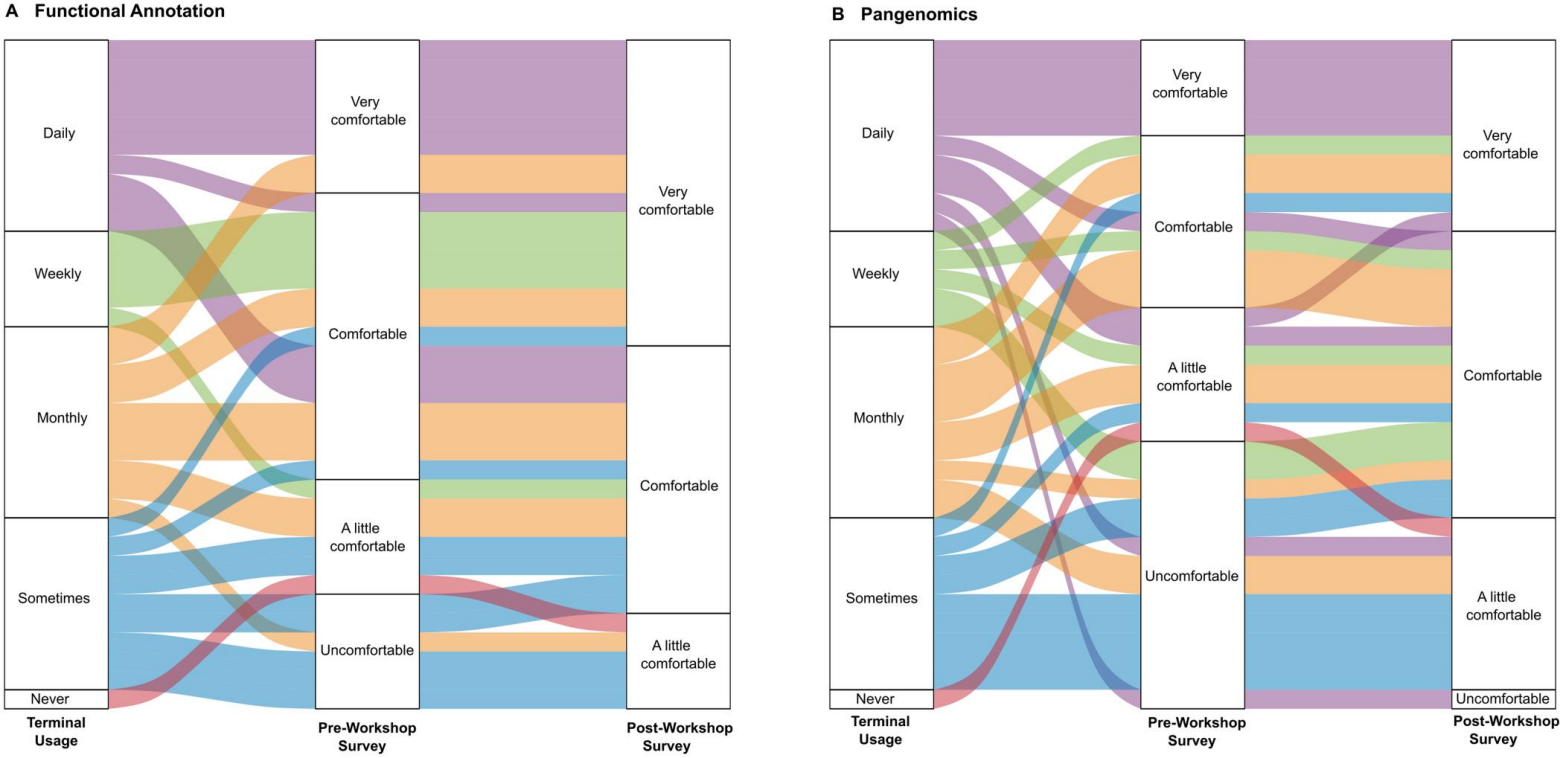
Comparison of pre- and post-workshop survey results on participant confidence in using functional annotation and Pangenomics tools. The column “Terminal Usage” indicates the frequency of terminal usage of the participants, while the second and third column indicate their corresponding level of confidence before and after the workshop on the topics “Functional Annotation” (A) and “Pangenomics” (B). The majority of participants increase their confidence in both topics.

When we taught the Pangenomics lesson through The National Bioinformatics Node, we launched a pre- and post-workshop survey to determine participants’ comfort and skill level in interacting with bioinformatics tools about Pangenomics. Regardless of their command line experience, attendees expressed a range of comfort levels with functional annotation and Pangenomics before the course. Following the course, their comfort levels notably improved, generally one level, ranging from a little comfortable to comfortable and very comfortable with Pangenomics (Fig. 4).

## 6 Conclusion

Our work facilitates Pangenomics analysis through specialized software for non-bioinformatics specialists. The workshop design follows the didactic principles of The Carpentries, providing a practical example from start to finish using real-world data. The selected data is highly relevant as it represents a set of genomes where variation in the presence and absence of genes was discovered, even among genomes of the same species. Additionally, we introduce another topic that lacks easily accessible tutorials: TDA.

Furthermore, the lesson is open source, allowing it to be utilized in a formal course or workshop. This collaborative effort within our practice community has been employed in various workshops, demonstrating the practical applicability of the introduced concepts.

## Supplementary Material

vbae139_Supplementary_Data

## Data Availability

The data underlying this article are available in Zenodo with DOI: 10.5281/zenodo.7620503 at https://zenodo.org/records/7974915.
