# Supplementary material for: Meeting the challenge of genomic analysis: a collaboratively developed workshop for pangenomics and topological data analysis
Source: Bioinform Adv. 2024 Sep 27;4(1):vbae139. doi: 10.1093/bioadv/vbae139 (PMC11525208; doi:10.1093/bioadv/vbae139)
Supplement: vbae139_Supplementary_Data [file vbae139_supplementary_data.pdf]

The following tables describe the exercises per episode in each lesson of the Worksohp. In Type column exercises are classified as follows: B stands for Begginer, I stands for Intermediate and A means Advanced. There are eleven exercises (5B/5I/1A) available for the lesson Introduction to the Command Line for Pangenomics distributed in 80 minutes. There are 10 (6B/3I/1A) exercises in the Introduction to Python distributed in 65 minutes. There are 15 (8B/5I/2A) exercises in the episodes of the Lesson Pangenome Analysis in Prokaryotes distributed in 110 minutes. There are 13 exercises (7B/5I/1A) in the Lesson Topological Data Analysis for Pangenomics distributed in 90 minutes. In the 16 hours of the workshop approximately a third (5hr and 45 min) are devoted to exercises whose difficulty is distributed in 26 begginer, 18 intermediate 5 advanced.

Table S1: Exercises in the six episodes of the Lesson Introduction to the Command Line for Pangenomics

Episode (Teaching minutes/ Exercise minutes)	Exercise name	Type
1. Introducing the Shell (20/5)	E1: Extra information with ‘ls -l‘	Open B
2. Navigating Files and Directories (30/15)	E1: Finding hiding directories<br>E2: Relative path resolution	Open B<br>Multiple choice I
3. Working with Files and Directories (30/10)	E1: Backup folder write-protected	Open I
4. Redirection (30/20)	E1: Using grep<br>E2: Using wc<br>E3: Using basename	Open B<br>Open I<br>Open A
5. Writing Scripts and Working with Data (20/15)	E1: Edit a file with nano<br>E2: Edit a script	Open B<br>Open I
6. Project Organization (15/15)	E1: Making an organized file system<br>E2: Creating a command’s record	Open B<br>Open I

Table S2: Exercises in the four episodes of the Lesson Introduction to the Python

Episode (Teaching minutes/ Exercise minutes)	Exercise name	Type
1. Introduction to Python (15/5)	E1: Creating variables and using functions	Open B
2. Data Structures (40/25)	E1: Manipulating lists<br>E2: Manipulating Dictionaries<br>E3: Manipulating arrays<br>E4: Manipulating dataframes	Open B<br>Multiple choice B<br>Multiple choice B<br>Open A
3. Functions (20/20)	E1: Evaluate if two strings have equal length<br>E2: Sort the function to count the nucleotides in a string<br>E3: Sort the comments to document a Hamming distance function for matrixes	Fill blanks B<br>Parson I<br>Fill comments I
4. Plotting (25/15)	E1: The nucleotide frequency of a DNA sequence<br>E2: Documenting a graph plot code	Fill blanks B<br>Fill comments I

Table S3: Exercises in the nine episodes of the Lesson Pangenome Analysis in Prokaryotes

Episode (Teaching minutes/ Exercise minutes)	Exercise name	Type
1. Introduction to Pangenomics. (10/15)	E1: Pizza Pangenomics<br>E2: Open or clossed	Discussion B<br>Discussion B
2. Downloading Genomic Data (30/15)	E1: Searching for desired strains	Select Answers B<br>Open A
3. Annotating Genomic Data (40/25)	E1: Inspecting the gbk<br>E2: Counting coding sequences<br>E3: The most abundant resistance mechanisms<br>E4: Annotating AMR of multiple genomes	False/True B<br>Open I<br>Fill blanks I<br>Fill blanks A
4. Measuring Sequence Similarity (30/10)	E1: Families in pizza pangenmics<br>E1: Remote blast search	Discussion B<br>Open I
5. Clustering with BLAST Results (30/5)	E1: Partitioning the Pangenome	Discussion B
6. Clustering Protein Sequences (30/10)	E1: Counting singletons	Fill blanks I
7. Exploring Pangenome Graphs (20/20)	E1: PpanGGolin results<br>E2: Exploring the pangenome graph	Discussion B<br>Open I
8.Interactive Pangenome Plots (20/10)	E1: Exploring the interactive plots.	Open B
9. Other resources (10/0)		

Table S4: Exercises in the six episodes of the Lesson Topological Data Analysis for Pangenomics

Episode (Teaching minutes/ Exercise minutes)	Exercise name	Type
1. Introduction to Topological Data Analysis (25/25)	E1: Identify the simplices<br>E2: Identify Betti numbers<br>E3: Calculate the Betti numbers<br>E4: Using the app example	Discussion B<br>Discussion B<br>Open I<br>Explore app A
2. Computational Tools for TDA (30/15)	E1: Creating a Manually Filtered Simplicial Complex<br>E2: Torus	Open I<br>Open I
3. Detecting horizontal gene transfer (30/30)	E1: Manipulating Dataframes (15/20)<br>E2: Plot persistent diagram<br>E3: Plot persistent barcode<br>E4: Is *S. agalactiae* resistome product of vertical inheritance?	Help B<br>Open B<br>Multiple choice B<br>Discussion I
4. Persistence Simplices gives rise to Gene Families (30/20)	E1: Partitioning the pangenome<br>E2: Looking for functional families<br>E3: Changing the dimension of the simplices	Open B<br>DiscussioinB<br>Open I
5. Other Resources (10/0)		
